# Left Ventricular Diastolic Dysfunction in Patients with Interstitial Lung Disease—A Potential Treatable Trait

**DOI:** 10.3390/arm94040057

**Published:** 2026-08-04

**Authors:** Ophir Freund, Uriel Katsoff, Tzlil Hershko, Ayala Ron, Shir Frydman, Doron Cohn-Schwartz, Aviv Kupershmidt, Neta Mano, Ariel Melloul, Eyal Kleinhendler, Amir Bar-Shai, Avraham Unterman

**Affiliations:** 1 Institute of Pulmonary Medicine, Sourasky Medical Center, Tel Aviv 6423906, Israelramiu@tlvmc.gov.il (A.U.); 2 Center of Excellence for Interstitial Lung Diseases, Tel Aviv Medical Center, Tel Aviv 6423906, Israel; 3 Gray Faculty of Medicine, Tel Aviv University, Tel Aviv 69978, Israel; 4 Cardiology Department, Tel Aviv Medical Center, Tel Aviv 6423906, Israel

**Keywords:** lung fibrosis, heart failure, prognosis, treatable traits, left ventricle dysfunction

## Abstract

**Highlights:**

**What are the main findings?**

- Left ventricular diastolic dysfunction (LVDD) has a 14% prevalence in patients with interstitial lung disease (ILD) and is concentrated in those with fibrotic disease phenotypes.
- In patients with fibrotic ILD, LVDD is an independent predictor of worse survival, a higher hazard for a composite adverse outcome (adjusted HR 1.82), and decreased exercise capacity.

**What is the implication of the main finding?**

- Because LVDD is reliably measurable via echocardiography and carries independent prognostic weight, it is a potentially important treatable trait in ILD.
- These findings emphasize the clinical need for systematic screening and close collaboration between pulmonologists and cardiologists to optimize personalized management in fibrotic ILD.

**Abstract:**

Cardiovascular comorbidities complicate interstitial lung disease (ILD) and represent potential therapeutic targets, yet the prevalence and clinical impact of left ventricular diastolic dysfunction (LVDD) remain poorly characterized. We aimed to evaluate LVDD as a potential treatable trait within a prospective cohort. Using data from an ILD registry (January 2021–October 2024), we defined clinically relevant LVDD as echocardiographic grade 2 or 3 diastolic dysfunction. Out of 276 patients (median age 69, 44% female), 14% had LVDD, which was almost entirely in patients with fibrosis (97%, *p* = 0.045) and associated with lower diffusing capacity for carbon monoxide. Survival and clinical analyses restricted to the fibrotic ILD subgroup (*n* = 241) showed that LVDD was independently associated with a higher hazard for a combined adverse outcome of acute exacerbation, lung transplantation, or death (adjusted HR 1.82, 95% CI 1.03–3.38, *p* = 0.043), which persisted after 1:2 propensity score matching (HR 2.04). LVDD also independently predicted increased all-cause mortality (adjusted HR 2.10) and reduced 6-min walk distance (median 413 vs. 465 m, β = −0.14, *p* = 0.036). In conclusion, LVDD is prevalent in fibrotic ILD and carries significant prognostic implications. Given its objective measurability and actionable treatment pathways, LVDD represents a potentially important treatable trait requiring multi-disciplinary care.

## 1. Introduction

Interstitial lung diseases (ILDs) are a heterogeneous group of chronic lung diseases that damage the lung interstitium through inflammation and abnormal wound-healing mechanisms, potentially leading to progressive lung fibrosis [1]. In recent years, positive outcomes from randomized controlled trials have led to the introduction of new drugs for many types of ILDs [2]. Nonetheless, ILDs impose a significant symptomatic burden and prognostic implications, considering that most drugs and interventions have only been shown to reduce the rate of lung function decline, with a large proportion of patients still having poor outcomes [3,4,5]. Therefore, a more comprehensive approach is needed to optimize disease management, such as the treatable trait approach. Treatable traits are defined as therapeutic targets that are reliably identified through a validated method and have demonstrated clinical relevance [6]. This approach extends beyond the focus on disease pathogenesis to incorporate phenotypes, endotypes, and patient factors to enable more personalized, comprehensive, and actionable treatments [7]. The wide heterogeneity in patient characteristics, disease etiology, and comorbidities among ILD patients highlights the potential of such an individualized approach to improve outcomes [8].

Cardiovascular diseases are considered a major extra-pulmonary treatable trait in patients with ILD [9]. Heart failure is one of the main comorbidities in patients with chronic lung diseases, often sharing similar symptoms and risk factors, resulting in delayed diagnosis and worse outcomes. Heart failure with preserved ejection fraction (HFpEF) accounts for over one-half of all heart failure cases [10]. The prominent finding in HFpEF is left ventricular diastolic dysfunction (LVDD), with several known risk factors such as obesity, hypertension, and diabetes [11]. Prior research on this entity in patients with ILD has primarily focused on idiopathic pulmonary fibrosis (IPF) and connective-tissue disease (CTD)-related ILDs [9], showing high rates of cardiovascular comorbidities. Still, most of these studies did not differentiate heart failure from other comorbidities [12,13], did not specify the heart failure type [14] or LVDD severity [15], and were limited by small sample sizes [16,17].

Considering the above, we aimed to assess the prevalence, predictors, and prognostic implications of clinically relevant LVDD in different types of ILD. We hypothesized that LVDD would be prevalent in a subset of ILD patients and would be associated with worse prognosis, highlighting a possible trait with available treatments.

## 2. Materials and Methods

This was an observational study using prospectively collected data from consecutive patients with ILD enrolled in a prospective registry between January 2021 and October 2024 at a large tertiary ILD center. Patients with available transthoracic echocardiographic (TTE) data and at least 1-year of follow-up were included in the study. All patients had an ILD diagnosis based on a multidisciplinary diagnosis (MDD), as previously described [18].

Data in the registry are collected at the first clinic visit (upon inclusion) and every 3–6 months, and include details on demographic characteristics, comorbidities, MDD diagnosis, pulmonary function, additional assessments (invasive procedures, TTE, etc.), and disease outcomes, as defined below. The presence of fibrotic disease was determined based on relevant radiological features, such as traction bronchiectasis or honeycombing, on chest high-resolution computed tomography (HRCT) as assessed by chest radiologists [19]. TTE was performed by specialized technicians, and results were recorded based on the final exam report. Of note, all patients in our clinic are routinely sent for TTE (if not recently performed) upon their first clinic visit, and the closest test to registry entry was included in the study.

The study was approved by the institutional review board (0456-21-TLV) and conducted in accordance with the Declaration of Helsinki. All patients signed informed consent to be enrolled in the prospective registry.

### 2.1. Study Definitions and Outcomes

Assessment of diastolic function was based on the American Society for Echocardiography guidelines [20,21]. In short, four primary variables were utilized for the initial screening of diastolic dysfunction: average septal and lateral mitral annular early diastolic velocity (e′ < 7 cm/s septal or <10 cm/s lateral), average E/e′ ratio (>14), left atrial volume index (LAVI > 34 mL/m^2^), and peak tricuspid regurgitation (TR) velocity (>2.8 m/s). Diastolic dysfunction was diagnosed if more than 50% of these parameters exceeded the defined thresholds. Specific grading relied primarily on the mitral inflow pattern, specifically the E/A ratio and E-wave velocity. LVDD was defined in our study as Grade II and III diastolic dysfunction. Pulmonary hypertension was defined as TTE-based pulmonary systolic pressure >40 mmHg.

The main study outcome was the prevalence of LVDD in patients with ILD. The disease adverse outcome was the occurrence of a combined outcome of ILD acute exacerbations (defined according to the International Working Group criteria across fibrotic ILD [22]), lung transplantation, and death. Additional outcomes included mortality and 6-min walk test distance (6MWD) using the test most adjacent to the TTE. Mortality was ascertained using the Ministry of Health national database, which is updated monthly.

### 2.2. Data Analysis

The sample size calculation was powered for the study’s main goal, assessing the prevalence of LVDD (Grade II and III) in patients with ILD. Prior research shows a range of LVDD prevalence in IPF patients [14]. We estimated the prevalence of LVDD at 15% across different types of ILD. Considering the estimated proportion, at least 196 patients with ILD were required to be included to achieve a 95% confidence level that the true LVDD prevalence would be within 5% of the measured value.

Continuous variables were presented as median (interquartile range) and categorical variables as counts (percentages of the total). Comparisons between patients with/without LVDD were performed using Mann–Whitney U tests for continuous variables and chi-square tests for categorical variables. The association between LVDD and disease outcomes was presented using Kaplan–Meier curves and analyzed using univariate and multivariate Cox regression analyses (including significant variables from the univariate model [*p* < 0.1] that were associated with LVDD, and that had clinical associations with the outcome, as determined by the research team). Association between LVDD and 6MWD was assessed using a multivariate linear regression model. A sensitivity analysis using propensity score matching (PSM) was performed between patients with and without LVDD. Matching included age, sex, obesity, heart failure, ejection fraction, pulmonary hypertension, history of ischemic heart disease, smoking status, and the subtype of ILD. PSM was performed using the nearest-neighbor algorithm with a ratio of 1:2 and a caliper distance of 0.10, with general linear models applied without replacement. All analyses were performed using SPSS (version 30.0, IBM Corp., Armonk, NY, USA).

## 3. Results

During the study period, the prospective registry consisted of 326 patients with ILD. Of them, 276 (85%) had available TTE results and were included in the study; 38 (14%) had LVDD. The cohort’s median (IQR) age was 70 (63–77), 44% were female, and 87% had a fibrotic ILD. The main ILD diagnoses included idiopathic pulmonary fibrosis (IPF, 20%), hypersensitivity pneumonitis (HP, 17%), and connective tissue-related ILD (CTD-ILD, 29%). Compared to the study population, patients without TTE results (who were excluded) were younger (median age 67 vs. 70, *p* = 0.016) and had a lower rate of fibrotic ILD (76% vs. 87%, *p* = 0.036), while other baseline characteristics were similar (Supplementary Table S1).

Comparison of the baseline characteristics between patients with and without LVDD is presented in Table 1. Patients with LVDD were older than those without (median [IQR] 76 [72–82] vs. 69 [62–76], *p* < 0.001) and had higher rates of clinician-assigned heart failure diagnosis in their medical record (32% vs. 12%, *p* < 0.001) and TTE-based pulmonary hypertension (42% vs. 17%, *p* < 0.001). All but one of the patients with LVDD had fibrotic ILD (97%), without differences in ILD diagnoses between the groups. Rates of anti-fibrotic therapy during follow-up were similar between the groups (21% for each). Patients with LVDD had lower DLCO (median 34 vs. 49% predicted) without differences in other pulmonary functions.

### 3.1. LVDD and ILD Outcomes

**Figure 1:**
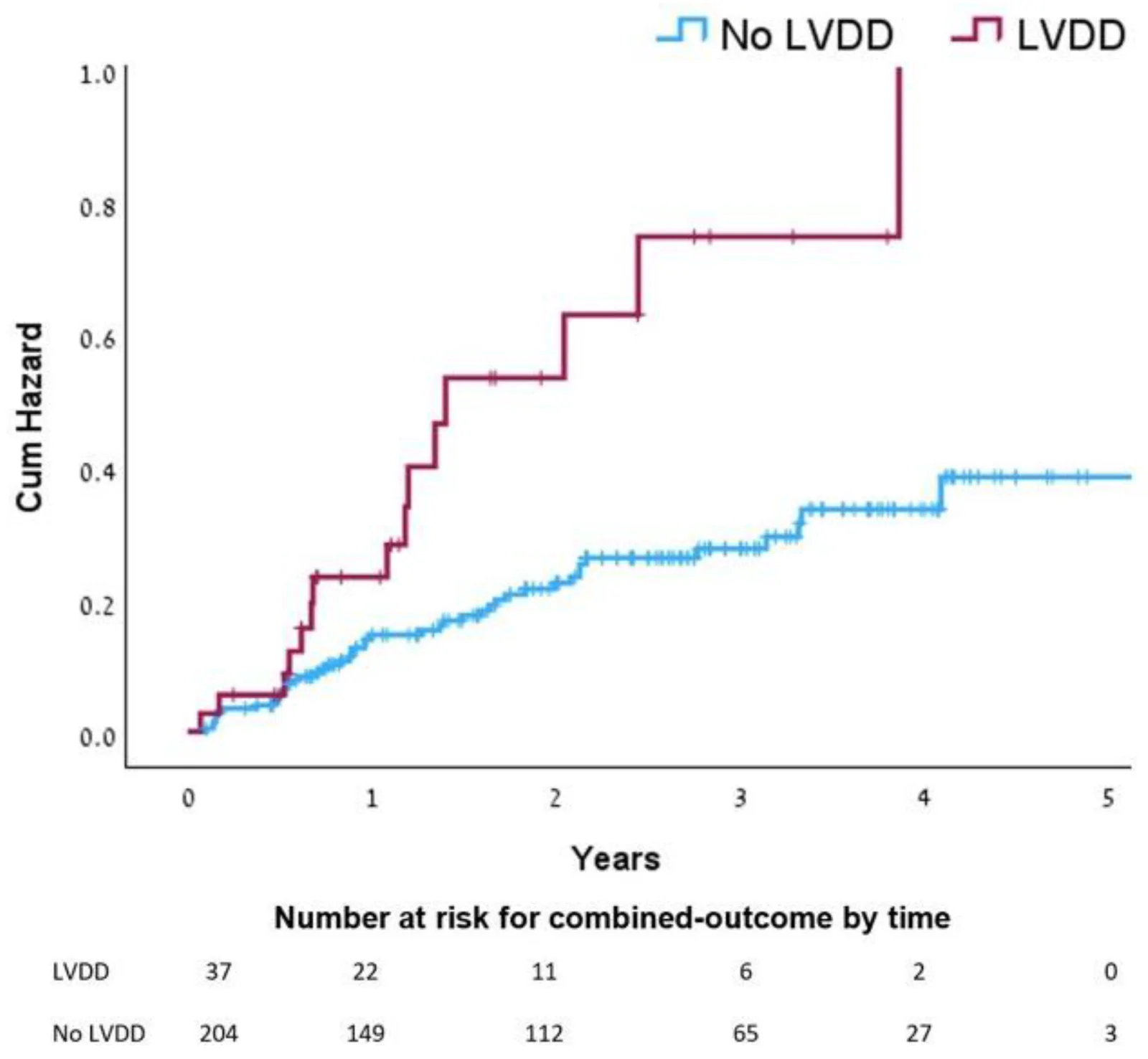
Kaplan–Meier curve for the combined outcome of ILD acute exacerbation, lung transplantation or death in patients with (red) or without (blue) LVDD (*p* = 0.003). Abbreviations: LVDD, left ventricular diastolic dysfunction.

Given the association of LVDD with fibrotic ILD (*p* = 0.045) and the rarity of non-fibrotic ILD in our cohort, we assessed the correlation between LVDD and ILD-related outcomes only among patients with fibrosis (*n* = 241). Associations between baseline characteristics and LVDD remained similar among fibrotic patients (Supplementary Table S2). The median (IQR) duration of follow-up was 2.1 (0.88–3.15) years. The combined outcome occurred in 60 patients (25%) during follow-up, including 15 in the LVDD group (41%) and 45 without LVDD (22%). Patients with LVDD had a higher hazard for the combined outcome (Figure 1), which remained significant in multivariate Cox regression analysis (Table 2, HR 1.82, 95% CI 1.03–3.38, *p* = 0.043). Increased age was also an independent predictor of the combined outcome (HR per additional year: 1.03; 95% CI, 1.004–1.06; *p* = 0.024).

**Figure 2:**
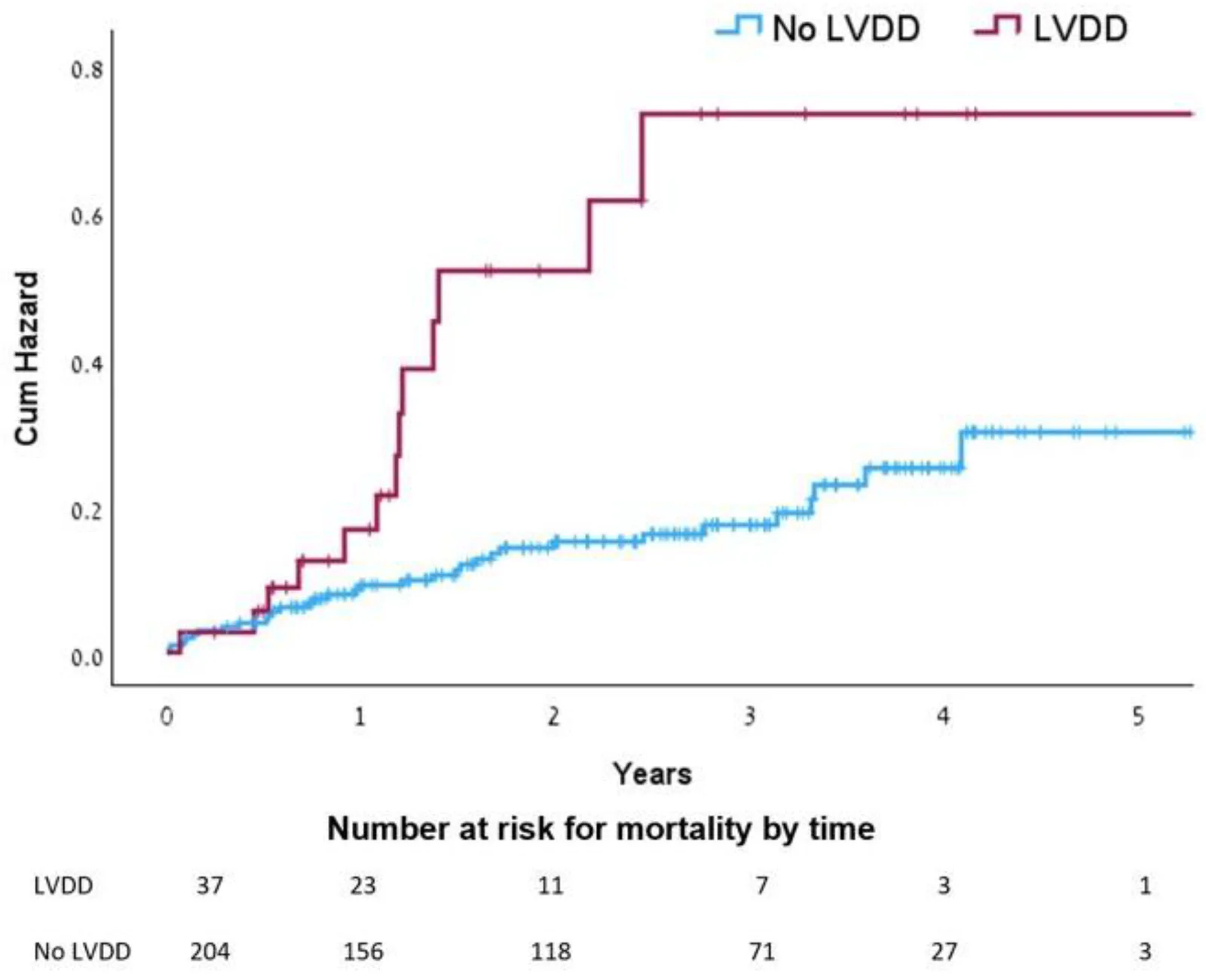
Kaplan–Meier curve for mortality in patients with (red) or without (blue) LVDD (*p* < 0.001). Abbreviations: LVDD, left ventricular diastolic dysfunction.

**Figure 3:**
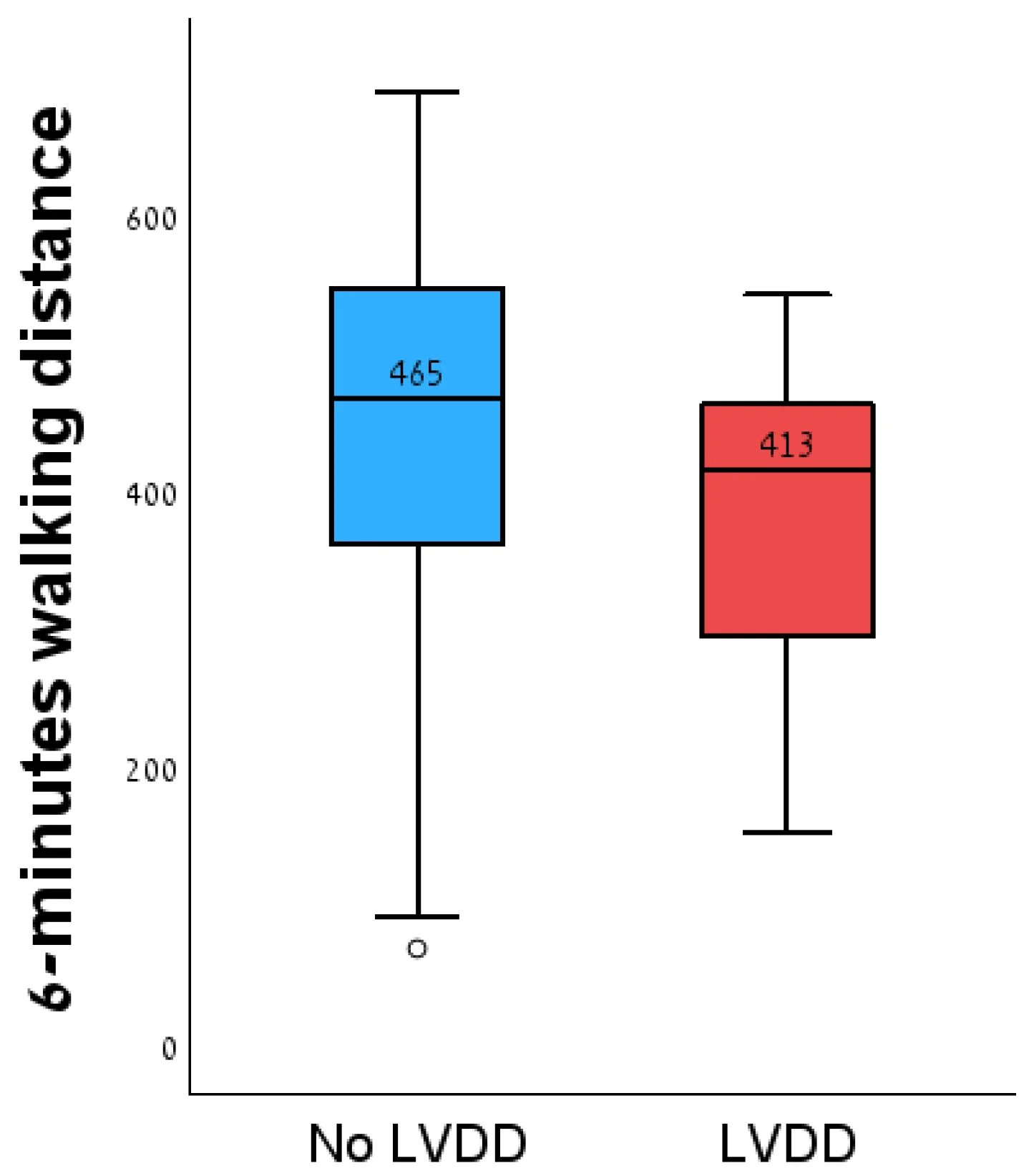
The 6-min walking test distance in patients with (red) and without (blue) left LVDD (*p* < 0.001). Abbreviations: LVDD, left ventricular diastolic dysfunction.

The presence of LVDD was associated with a higher hazard of mortality (Figure 2), which persisted in a multivariate Cox regression analysis (Table 2; HR 2.10, 95% CI 1.06–4.16, *p* = 0.033). TTE-based pulmonary hypertension was also associated with mortality. 6MWD results were available for 186 patients (77%). Patients with LVDD had a lower 6MWD distance (Figure 3, median [IQR] 413 [289–462] vs. 465 [360 vs. 544] meters, *p* < 0.001). LVDD remained a predictor of lower 6MWD in a multivariate linear regression analysis (Table 3, β = −0.14, *p* = 0.036). Older age was also associated with a lower 6MWD distance.

### 3.2. Matched Cohorts

**Figure 4:**
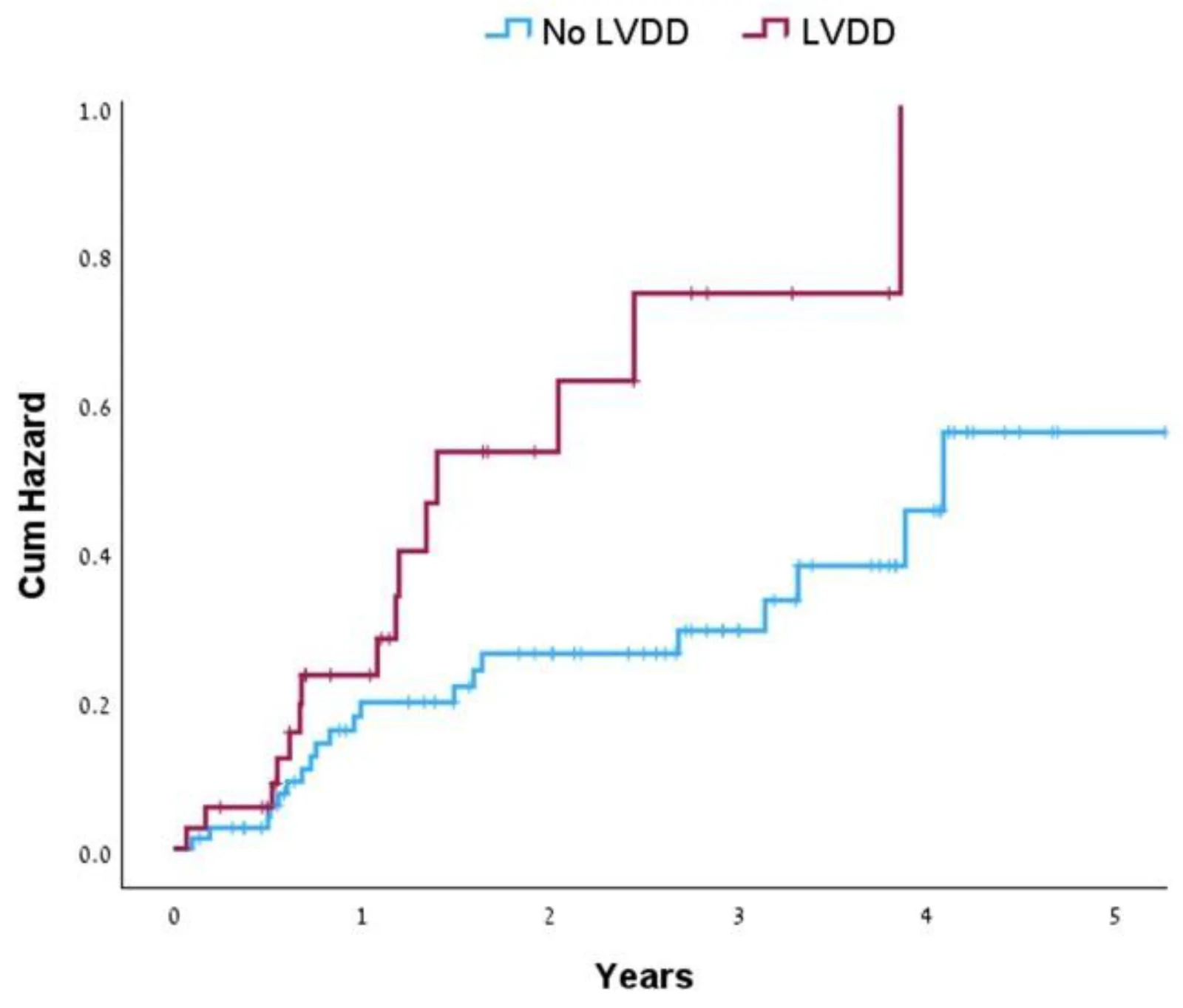
Kaplan–Meier curve for the combined outcome following propensity score matching in patients with (red) or without (blue) LVDD (*p* = 0.039). Abbreviations: LVDD, left ventricular diastolic dysfunction.

We validated our results by performing propensity score matching among patients with fibrosis, using a 1:2 ratio of those with and without LVDD, as described in the Methods section. Following matching, there was no difference between groups in any baseline characteristics (Supplementary Table S3). LVDD remained associated with higher hazards for the combined outcome (Figure 4, HR 2.04, 95% CI 1.04–4.00, *p* = 0.039) and for mortality (HR 2.56, 95% CI 1.19–5.47, *p* = 0.016, Supplementary Figure S1). The 6MWD was not significantly lower in the LVDD group following matching (median [IQR] 413 [289–462] vs. 435 [360 vs. 477] meters, *p* = 0.068).

## 4. Discussion

In this analysis of prospectively collected data of patients with ILD, LVDD was prevalent, especially among those with fibrotic disease. Older age was the only baseline characteristic associated with LVDD, with lower DLCO and higher pulmonary artery systolic pressure as additional predictors. Finally, LVDD was a predictor of worse disease outcomes, an association that persisted in multivariate models and after propensity score matching.

In general, for a feature to be considered a trait for a specific disease, it should meet three core criteria [23]. First, it should be clinically important. Our results underscore the clinical implications of LVDD in ILD patients and are in line with previous findings from specific ILD subtypes [14,24]. Second, the feature needs to be recognizable and measurable. In the case of LVDD, not only is TTE a known and validated diagnostic method, but known predictors for its presence could facilitate early identification, such as low DLCO [25] and increased pulmonary systolic pressure [24]. Third, the feature must be treatable. In recent years, several therapies have been shown to have a beneficial effect in patients with HFpEF, including SGLT2 inhibitors [26], the mineralocorticoid receptor antagonist spironolactone [27], and the angiotensin receptor-neprilysin inhibitor sacubitril/valsartan [28]. We do not suggest that all patients with LVDD have HFpEF, although this must be considered in these patients. In addition, the identification and management of known risk factors for LVDD, such as hypertension and obesity, might prevent the development of more severe LVDD [29].

Even though LVDD meets the above criteria for consideration as a treatable trait in patients with ILD, it is still not recognized as such by some [7,9]. Higher awareness among physicians is crucial to consider this as a possible etiology of progressive disease and to facilitate early diagnosis and treatment. It is important to note that while LVDD is a cardiac imaging finding, HFpEF is the main clinical syndrome resulting from it. The clinical diagnosis of HFpEF requires an ejection fraction ≥50% and signs and symptoms not attributable to another condition [30]. This definition highlights the need for a multidisciplinary team for patients with concurrent ILD and LVDD, including HF specialists to assess the presence of HFpEF and provide personalized treatment. Although guidelines recommend echocardiographic evaluation in patients with lung disease when pulmonary hypertension is suspected, no specific recommendations exist regarding screening for LVDD in patients with ILD. Based on our findings, a pragmatic approach could be considered whereby echocardiography is performed in patients with unexplained dyspnea, disproportionate reduction in exercise capacity or DLCO, or physiological deterioration despite otherwise stable ILD. Patients with moderate or severe LVDD should subsequently undergo cardiology evaluation including specialist referral. These suggestions should be considered hypothesis-generating and require prospective validation before being incorporated into clinical practice.

The prevalence of LVDD in the general population is estimated between 7% and 19% in various studies [31,32]. The prevalence in our cohort (15%) is on the higher side of these rates, with several possible explanations. Older age is an important risk factor for both fibrotic ILD and LVDD. Other known risk factors for LVDD in the general population were also prevalent in our cohort. While they were not found to be associated with LVDD, they could still contribute to its development. Subclinical or underdiagnosed coronary vascular disease is known to be highly prevalent among ILD patients [33], with estimates of up to 60%, and is a main contributor to LVDD [9]. Coronary microvascular disease is another etiology for LVDD with high prevalence among prior or current smokers [34,35]. Pulmonary hypertension also contributes to LVDD, which could be the result of distortion of the interventricular septum towards the left ventricle as the right ventricle adapts to pressure or volume overload [17]. Progressive right ventricular pressure overload may impair left ventricular filling through ventricular interdependence and reduced left ventricular preload, while elevated left-sided filling pressures may further exacerbate pulmonary vascular congestion. This bidirectional cardiopulmonary interaction may partly explain why both pulmonary hypertension and LVDD independently predicted adverse outcomes in our cohort. A different explanation for the relatively high prevalence of LVDD in patients with fibrotic ILD might be a shared pulmonary and cardiac fibrotic pathological process. Lewandowski et al. assessed cardiac fibrosis via cardiac MRI in 549 patients and found LVDD to be correlated with higher rates of cardiac scarring [36]. Systemic inflammation is known to be a driver in some cases of ILD and LVDD, as previously shown in patients with CTD-ILD [37]. Finally, pirfenidone, an anti-fibrotic agent with proven efficacy for IPF [38], has also been shown to reduce cardiac fibrosis (defined by extracellular volume) compared with the placebo [39]. Still, more evidence is needed, especially given the worse outcomes shown in ILD patients with some of the treatments for HFpEF [40,41]. Future studies should move beyond observational associations and incorporate multimodal mechanistic approaches. Systems biology may be particularly valuable by integrating clinical data with imaging, circulating biomarkers, genomics, transcriptomics, proteomics, and metabolomics to identify shared molecular pathways underlying pulmonary and myocardial fibrosis. Such approaches may clarify whether LVDD and fibrotic ILD represent parallel manifestations of common biological processes, or whether the association is predominantly mediated by secondary hemodynamic changes such as pulmonary hypertension. Longitudinal studies combining serial echocardiography, advanced cardiac imaging, and molecular profiling may further help distinguish causal mechanisms from disease progression and identify patients most likely to benefit from targeted cardiovascular therapies.

The main strengths of our study are the relatively large sample size, prospective data collection, and multiple validation strategies for the associations with long-term outcomes. The study also includes some important limitations. First, the registry is from a single tertiary expert ILD center, limiting its generalizability. It could also lead to a referral bias with more complex cases. Second, the TTE examinations were not performed for the purposes of the study and were not standardized in terms of equipment and operators, which may have introduced inter-observer variability in the assessment of diastolic function. Third, only specific TTE variables were included in our registry, resulting in missing data for most patients on the variables the technician used to grade LVDD. This has also led to the definition of pulmonary hypertension based on SPAP alone, given that most patients did not undergo right heart catheterization. Fourth, we collected data only on all-cause mortality; therefore, we could not address associations with ILD-related or cardiovascular mortality, limiting the ability to determine whether the observed prognostic effect is directly mediated by cardiovascular events. Fifth, assessment of cardiovascular treatments was beyond the study’s scope; even if provided, LVDD remained a strong prognostic finding. Finally, residual confounding by baseline ILD severity cannot be completely excluded. However, apart from DLCO, the groups were comparable with respect to other established markers of disease severity, including FVC, TLC, fibrotic ILD subtype, radiological fibrosis, and initiation of antifibrotic therapy. Moreover, because DLCO is influenced not only by the severity of fibrotic lung disease, but also by pulmonary hypertension and left-sided cardiac dysfunction, it should be interpreted as an integrated marker of pulmonary and cardiovascular impairment rather than a measure of ILD severity alone.

## 5. Conclusions

LVDD is a prevalent comorbidity in patients with fibrotic ILD, associated with reduced lung function and exercise capacity, and carries significant prognostic implications. The available treatment options and preventive measures make LVDD a potentially important treatable trait in patients with ILD, requiring higher awareness and collaboration between pulmonologists and cardiologists to improve patient care and outcomes.

## Figures and Tables

**Table 1:** Comparison of baseline characteristics between patients with and without LVDD.

Variable	LVDD *N* = 38 (%)	No LVDD *N* = 238 (%)	*p*
Age	76 (72–82)	69 (62–76)	<0.001
Female sex	18 (47)	103 (43)	0.637
Obese (BMI > 30)	8 (21)	35 (15)	0.316
Smoking status			
Never	16 (42)	92 (39)	0.777
Prior	20 (53)	126 (53)	
Current	2 (5)	20 (8)	
GERD	14 (37)	114 (48)	0.196
Heart failure diagnosis	12 (32)	28 (12)	0.001
Ischemic heart disease	9 (24)	37 (16)	0.211
Hyperlipidemia	18 (47)	110 (46)	0.895
Hypertension	17 (45)	101 (42)	0.790
Diabetes	10 (26)	60 (25)	0.884
Echocardiographic results			
Ejection fraction, %	60 (55–60)	60 (60–60)	0.498
Ejection fraction < 40%	2 (5)	6 (3)	0.303
PASP, mmHg	37 (32–55)	32 (27–38)	<0.001
PASP > 40 mmHg	16 (42)	40 (17)	<0.001
Moderate aortic stenosis ^a^	1 (4)	3 (1)	0.504
Moderate aortic regurgitation ^a^	1 (4)	3 (1)	0.504
Moderate mitral stenosis ^a^	0 (0)	1 (0)	1.00
Moderate mitral regurgitation ^a^	2 (7)	6 (2)	0.250
Disease-related variables			
Fibrotic ILD	37 (97)	204 (86)	0.045
ILD diagnosis			
Idiopathic pulmonary fibrosis	5 (12)	50 (21)	0.758
Hypersensitivity pneumonitis	9 (24)	37 (16)	
CTD-ILD	13 (34)	66 (28)	
Unclassifiable	8 (21)	44 (19)	
Other	3 (8)	41 (17)	
Initiation of anti-fibrotic therapy during follow-up	8 (21%)	51 (21%)	0.958
FEV1, %predicted	77 (57–96)	80 (68–91)	0.697
FVC, %predicted	73 (62–91)	76 (64–88)	0.582
FEV1/FVC	0.82 (0.75–0.88)	0.83 (0.77–0.88)	0.392
TLC, %predicted ^b^	75 (68–89)	81 (68–92)	0.363
DLCO, %predicted ^b^	34 (28–41)	49 (36–64)	<0.001

Abbreviations: CTD, connective tissue disease; DLCO, diffusing capacity for carbon monoxide; FEV1, Forced Expiratory Volume in 1 Second; FVC, forced vital capacity; GERD, gastroesophageal reflux disease; ILD, interstitial lung disease; LVDD, left ventricular diastolic dysfunction; TLC, total lung capacity. ^a^ There were no cases of severe valvular dysfunction. ^b^ Data available for 221 patients (80%).

**Table 2:** Multivariate Cox regression for the study outcomes.

Variable	Univariate		Multivariate	
	HR (95% CI)	*p*	Adjusted HR (95% CI)	*p*
Combined outcome				
Age, for 1 additional year	1.04 (1.02–1.07)	0.002	1.03 (1.004–1.06)	0.024
LVDD	2.45 (1.36–4.40)	0.003	1.82 (1.03–3.38)	0.043
Pulmonary hypertension	2.41 (1.43–4.08)	0.001	1.29 (0.69–2.41)	0.423
Heart failure diagnosis	1.95 (1.09–3.50)	0.025	1.66 (0.93–2.97)	0.088
Mortality				
Age, for 1 additional year	1.04 (1.01–1.08)	0.009	1.03 (0.99–1.06)	0.118
LVDD	3.06 (1.60–5.86)	<0.001	2.10 (1.06–4.16)	0.033
Pulmonary hypertension	3.36 (1.87–6.05)	<0.001	2.19 (1.14–4.22)	0.019
Heart failure diagnosis	2.62 (1.39–4.93)	0.003	1.57 (0.80–3.09)	0.194

Abbreviations: LVDD, left ventricular diastolic dysfunction.

**Table 3:** Multivariate linear regression of predictors for the 6-min walking test distance.

Variable	Coefficient	Standard Error	Beta	t	*p* Value
Age, per 1 additional year	−5.40	0.869	−0.420	−6.205	<0.001
LVDD	−52.14	24.66	−0.143	−2.114	0.036
Pulmonary hypertension	−7.74	24.26	−0.023	−0.319	0.750
Heart failure diagnosis	−19.93	25.32	−0.055	−0.787	0.432

Abbreviations: LVDD, left ventricular diastolic dysfunction.

## Data Availability

All data generated or analyzed during this study are included in this article. Due to ethical and privacy concerns, the primary dataset cannot be made openly available. Request for the dataset supporting our results can be made via helsinki@tlvmc.gov.il and will be given by the first author after approval.

## Supplementary Materials

The following supporting information can be downloaded at: https://www.mdpi.com/article/10.3390/arm94040057/s1. Table S1, Comparison of baseline characteristics between those with (included) and without (excluded) available echocardiography results. Table S2, Comparison of baseline characteristics in fibrotic disease between patients with and without LVDD. Table S3, Comparison of baseline characteristics following propensity score matching between patients with and without LVDD. Figure S1, Kaplan-Meier curve for mortality in patients with (red) or without (blue) LVDD (*p* = 0.016) following propensity score matching. Abbreviations: LVDD, left ventricular diastolic dysfunction.
