# Supplementary material for: Left Ventricular Diastolic Dysfunction in Patients with Interstitial Lung Disease—A Potential Treatable Trait
Source: Adv Respir Med. 2026 Aug 4;94(4):57. doi: 10.3390/arm94040057 (PMC13510215; doi:10.3390/arm94040057)
Supplement: Supplementary file 1 [file arm-94-00057-s001.zip › arm-4453105-supplementary.pdf]

Supplementary

Left ventricular diastolic dysfunction in patients with interstitial lung disease - a potential treatable trait

Table S1, Comparison of baseline characteristics between those with (included) and without (excluded) available echocardiography results.

Table S2, Comparison of baseline characteristics in fibrotic disease between patients with and without LVDD.

Table S3, Comparison of baseline characteristics following propensity score matching between patients with and without LVDD.

Figure S1, Kaplan-Meier curve for mortality in patients with (red) or without (blue) LVDD (p=0.016) following propensity score matching. Abbreviations: LVDD, left ventricular diastolic dysfunction.

Table S1, Comparison of baseline characteristics between those with (included) and without (excluded) available echocardiography results.

**Variable**	**Included N=276 (%)**	**Excluded N=50 (%)**	**p**
Age	70 (63-77)	67 (59-71)	0.016
Female sex	121 (44)	28 (56)	0.112
Smoking status			
Never	108 (39)	21 (42)	
Prior	146 (53)	21 (42)	0.133
Current	22 (8)	8 (16)	
Heart failure	40 (15)	5 (10)	0.397
Ischemic heart disease	46 (17)	7 (14)	0.638
Hyperlipidemia	128 (46)	16 (32)	0.060
Hypertension	118 (43)	16 (32)	0.155
Diabetes	70 (25)	11 (22)	0.613
Fibrotic ILD	241 (87)	38 (76)	0.036
ILD Diagnosis			
Idiopathic pulmonary fibrosis	55 (20)	9 (18)	
Hypersensitivity pneumonitis	46 (17)	9 (18)	
CTD-ILD	79 (29)	11 (22)	0.830
Other	52 (19)	9 (18)	
Unclassifiable	44 (16)	12 (24)	
FVC, %predicted	76 (64-89)	81 (67-91)	0.280
TLC, %predicted	80 (68-93)	85 (71-97)	0.301
DLCO, %predicted	48 (38-63)	53 (40-77)	0.064

Abbreviations: CTD, connective-tissue disease; DLCO, diffusing capacity for carbon monoxide; FVC, forced vital capacity; ILD, interstitial lung disease; LVDD, total lung capacity.

Table S2, Comparison of baseline characteristics in fibrotic disease between patients with and without LVDD.

**Variable**	**LVDD N=37 (%)**	**No LVDD N=204 (%)**	**p**
Age	76 (68-82)	71 (64-77)	0.007
Female sex	17 (46)	86 (42)	0.668
Obese (BMI>30)	7 (19)	31 (15)	0.568
Smoking status			
Never	16 (42)	76 (37)	
Prior	20 (53)	113 (55)	0.516
Current	1 (3)	15 (7)	
GERD	14 (38)	100 (49)	0.201
Heart failure	11 (30)	25 (12)	0.006
Ejection fraction, %	60 (55-60)	60 (60-60)	0.497
Ischemic heart disease	9 (24)	35 (17)	0.299
Pulmonary hypertension	16 (43)	36 (18)	<0.001
Hyperlipidemia	18 (49)	97 (48)	0.902
Hypertension	16 (43)	88 (43)	0.990
Diabetes	9 (24)	58 (28)	0.608
Disease-related variables			
ILD Diagnosis			
Idiopathic pulmonary fibrosis	5 (14)	50 (24)	
Hypersensitivity pneumonitis	8 (22)	32 (16)	
CTD-ILD	13 (35)	60 (29)	0.554
Other	3 (8)	24 (12)	
Unclassifiable	8 (22)	38 (19)	
Initiation of anti-fibrotic agent during follow-up	8 (22)	51 (25)	0.660
FEV1, %predicted	76 (57-96)	80 (68-91)	0.527
FVC, %predicted	72 (62-91)	75 (64-87)	0.637
FEV1/FVC	0.81 (0.75-0.87)	0.84 (0.78-0.90)	0.103
TLC, %predicted*	74 (67-89)	78 (66-91)	0.631
DLCO, %predicted*	39 (35-47)	51 (39-63)	0.003

Abbreviations: CTD, connective-tissue disease; DLCO, diffusing capacity for carbon monoxide; FEV1, Forced Expiratory Volume in 1 Second; FVC, forced vital capacity; GERD, gastro-esophageal reflux disease; ILD, interstitial lung disease; LVDD, left-ventricular diastolic dysfunction; TLC, total lung capacity.

* Data available for 198 patients (82%).

Table S3, Comparison of baseline characteristics following propensity score matching between patients with and without LVDD.

**Variable**	**LVDD N=37 (%)**	**No LVDD N=74 (%)**	**P**
Age	76 (68-82)	76 (72-81)	0.409
Female sex	17 (46)	25 (34)	0.213
Obese (BMI>30)	7 (19)	14 (19)	1.000
Smoking status			
Never	16 (42)	28 (37)	
Prior	20 (53)	44 (60)	0.858
Current	1 (3)	2 (3)	
GERD	14 (38)	34 (47)	0.383
Heart failure	11 (30)	25 (34)	0.667
Ejection fraction, %	60 (55-60)	60 (55-60)	0.636
Ischemic heart disease	9 (24)	25 (34)	0.308
Pulmonary hypertension	16 (43)	36 (48)	0.591
Hyperlipidemia	18 (49)	40 (54)	0.591
Hypertension	16 (43)	42 (57)	0.179
Diabetes	9 (24)	58 (28)	0.155
Disease-related variables			
ILD Diagnosis			
Idiopathic pulmonary fibrosis	5 (14)	22 (30)	
Hypersensitivity pneumonitis	8 (22)	10 (14)	
CTD-ILD	13 (35)	14 (19)	0.123
Other	3 (8)	11 (15)	
Unclassifiable	8 (22)	17 (23)	
FEV1, %predicted	76 (57-96)	80 (67-91)	0.538
FVC, %predicted	72 (62-91)	75 (64-87)	0.705
FEV1/FVC	0.81 (0.75-0.87)	0.83 (0.78-0.90)	0.319
TLC, %predicted*	74 (67-89)	77 (65-88)	0.901
DLCO, %predicted*	39 (35-47)	45 (33-58)	0.223

Abbreviations: CTD, connective-tissue disease; DLCO, diffusing capacity for carbon monoxide; FEV1, Forced Expiratory Volume in 1 Second; FVC, forced vital capacity; GERD, gastro-esophageal reflux disease; ILD, interstitial lung disease; LVDD, left-ventricular diastolic dysfunction; TLC, total lung capacity.

* Data available for 93 patients (84%).

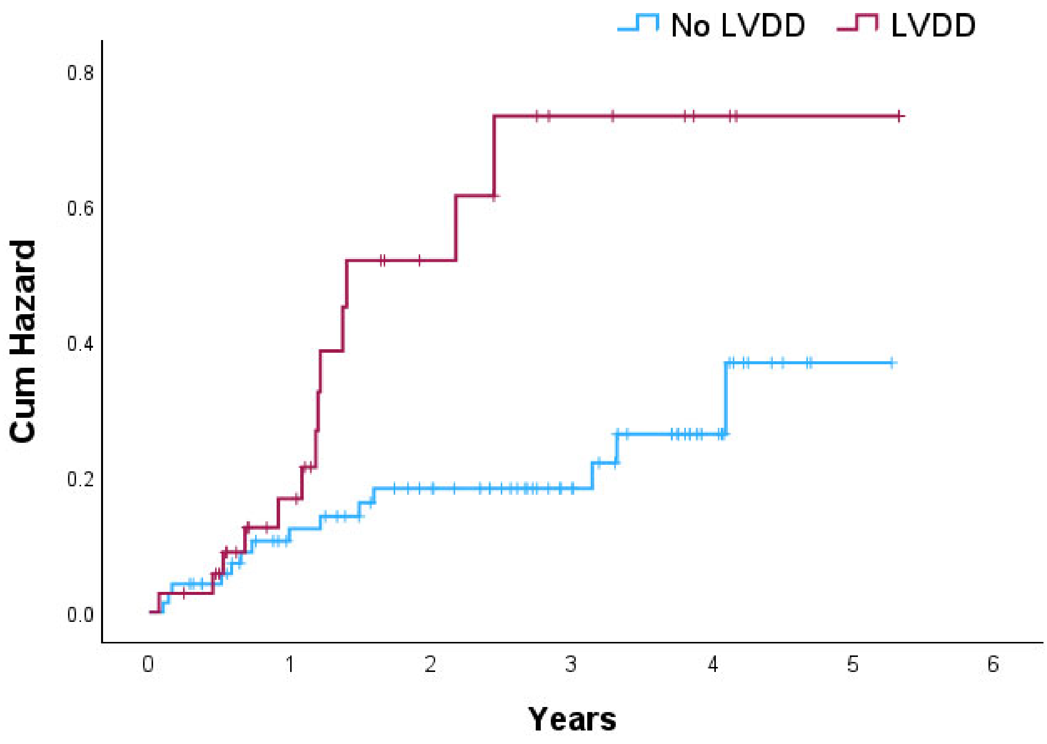

Figure S1, Kaplan-Meier curve for mortality in patients with (red) or without (blue) LVDD (p=0.016) following propensity score matching. Abbreviations: LVDD, left ventricular diastolic dysfunction.
